# ROS-based ground stereo vision detection: implementation and experiments

**DOI:** 10.1186/s40638-016-0046-y

**Published:** 2016-09-02

**Authors:** Tianjiang Hu, Boxin Zhao, Dengqing Tang, Daibing Zhang, Weiwei Kong, Lincheng Shen

**Affiliations:** College of Mechatronics and Automation, National University of Defense Technology, Changsha, 410073 People’s Republic of China

**Keywords:** Unmanned aerial vehicle (UAV), Autonomous landing, Robot operating systems (ROS), Chan–Vese, Flying object detection

## Abstract

This article concentrates on open-source implementation on flying object detection in cluttered scenes. It is of significance for ground stereo-aided autonomous landing of unmanned aerial vehicles. The ground stereo vision guidance system is presented with details on system architecture and workflow. The Chan–Vese detection algorithm is further considered and implemented in the robot operating systems (ROS) environment. A data-driven interactive scheme is developed to collect datasets for parameter tuning and performance evaluating. The flying vehicle outdoor experiments capture the stereo sequential images dataset and record the simultaneous data from pan-and-tilt unit, onboard sensors and differential GPS. Experimental results by using the collected dataset validate the effectiveness of the published ROS-based detection algorithm.

## Background

In the past decades, unmanned aerial vehicles (UAVs) have been widely used in many fields. The applications include environmental monitoring, planting and farming, remote observation and earthquake rescue [1]. Most attention is generally paid on fixed-wing aerial vehicle recovery because of relatively higher risk involved during the landing phase. Many practical applications showed that recovery is the most challenging and hazardous period of UAV flights [2]. Developing autonomous landing technologies has already been an important trend of runway-mode takeoff-and-landing UAV systems. It aims at reducing personnel dependency and workload and meanwhile improving adaptability and reliability of flying vehicles recovery. The success of flying aircraft navigation is mostly achieved by using onboard conventional sensors, such as global positioning system (GPS), inertial measurement unit (IMU) and magnetometer. However, autonomous landing task that requires higher accuracy in localization is still not achievable solely by these onboard sensors [3, 4].

Under such circumstances, a ground vision guidance scheme was proposed and developed [5–11]. The ground system possesses stronger computation resources and saves cost by implementing each set for a runway rather than individual vehicles. Moreover, image processing on the ground-captured images is more convenient than that on the onboard images with complicated backgrounds.

**Fig. 1:**
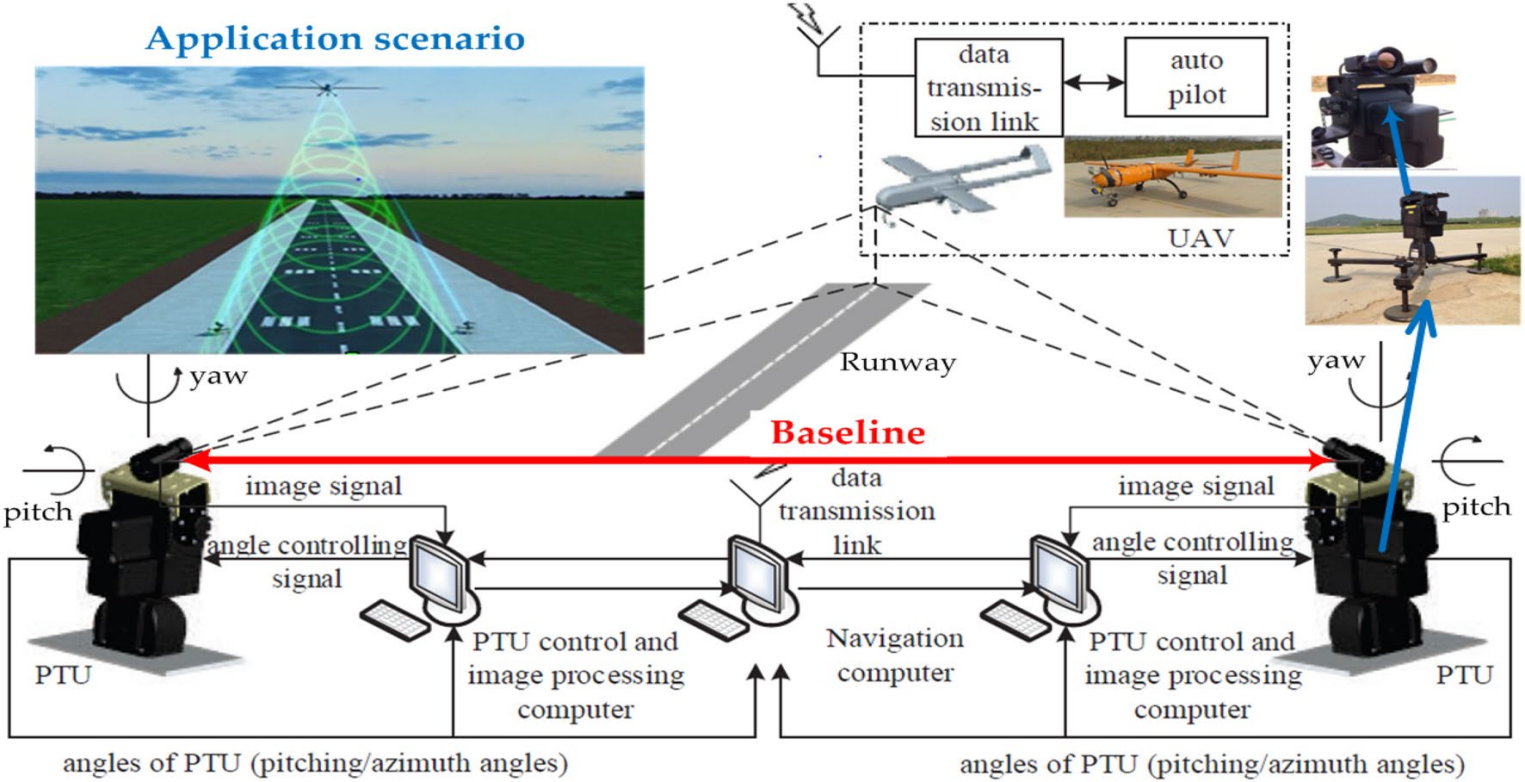
Architecture and scenarios of the ground stereo vision guidance and localization system. The binocular cameras are located symmetrically on both sides of the runway to capture sequential images of the approaching and landing unmanned vehicles. The ground system with stronger processing abilities calculates the spatial localization and sends the coordinates onto the onboard autopilot (adopted from Tang and Hu et al. [8, 9])

Runway landing and taxiing has been a kernel recycling mode of medium and/or large fixed-wing unmanned aerial vehicles. Vision-based localization and guidance has drawn more and more attention in the field of UAV autonomous takeoff and landing [12]. Hereafter, a ground stereo vision guidance system has been proposed and presented [7–9]. As shown in Fig. 1, the binocular cameras are located symmetrically on both sides of the runway to capture sequential images of the approaching and landing unmanned aircrafts. The ground system with stronger processing abilities calculates the spatial coordinates by integrating calibration, detection and localization steps. Eventually, the ground system sends the coordinates into the onboard autopilot via the specified data link. In our previous works [6–9], both corner-based and skeleton-based algorithms were employed into the flying object detection on the ground-captured sequential images. As for the corner-based methods, Harris [13], SIFT [14], SURF [15], ORB [16], FAST [17] and BRISK [18] corner detectors are, respectively, tested with the dataset. As for the skeleton-based methods, level set, Canny and Chan–Vese [19] are generally employed into the edge extraction.

In this study, a synthetic data-driven scheme is developed and presented for target detection algorithm design, implementation, testing, evaluation and parameter tuning. The Chan–Vese [19] approach is demonstrated as a case study. The Chan–Vese object detection algorithm is to be implemented in the robot operating system (ROS) platform for general multi-user usages, open-source support and inheritable development. The dataset of stereo sequential images is constructed to evaluate detection performance and to tune appropriate parameters as well. The ROS package is developed and published on the open-source *github* Web site. The comparisons are made between the Chan–Vese automatic detection and the manual detection based on the collected dataset. The results show that the ROS-based Chan–Vese detection approach effectively extracts the aircraft coordinates with satisfied localization accuracy.

## System architecture and workflow

### Architecture of ground stereo vision system

Aerial vehicles autonomous landing on the runway is usually composed of three stages: approaching, descending and taxiing. The onboard navigation system guides the aircraft into the field of view of stereo cameras. Once the aircraft target is detected, the spatial coordinates are calculated by using the stereo vision localization algorithm. The data link connects the flying aircraft and the ground system and transfers the vision-based localized position onto the onboard autopilot. Detailed process and scenarios are presented in Fig. 1.

### Stereo localization workflow

The ground stereo vision system consists of two independent modules. Each module is equipped with one camera on an independent pan–tilt unit. The two modules are independently connected to the computer. Landing image sequences are obtained by the symmetrically located two cameras on both sides of the runway. The pan–tilt units are automatically driven to keep the flying aircraft around the center of the vision field. The pan-and-tilt angles are fed back to the computer for calculating the spatial coordinates.

**Fig. 2:**
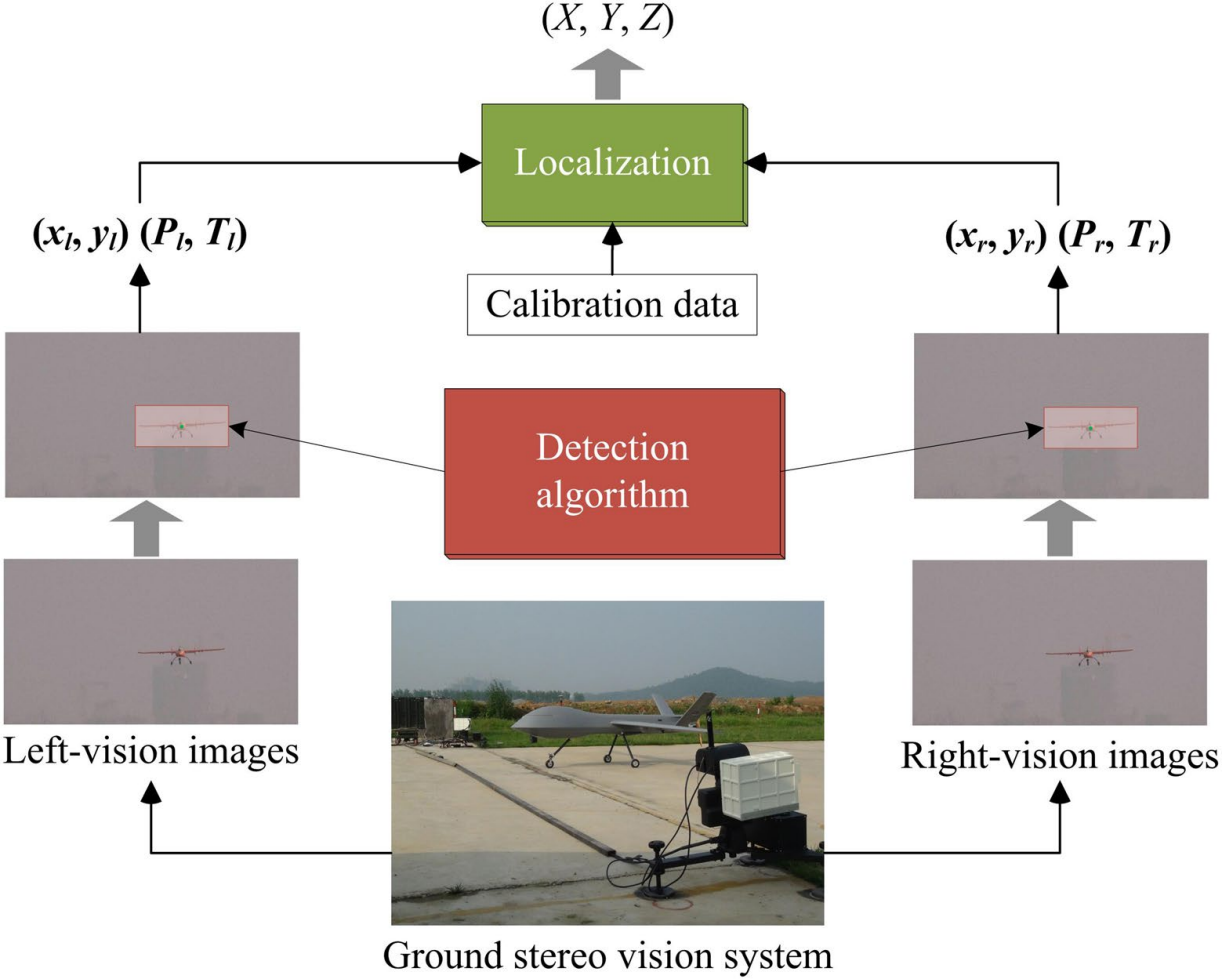
Algorithm workflow of the ground stereo vision guidance and localization system. Stereo vision guidance system mainly consists of image capture, aircraft detection and tracking, and localization. Detection algorithm transfers the captured images into a pair of pixel points standing for the extracted object positions (*x*_*l*_, *y*_*l*_) and (*x*_*r*_, *y*_*r*_). Localization algorithm generates the spatial coordinates by fusing the calibration data, a pair of detected pixel points and the feedback angles of pan–tilt units

The ground detection component usually works through the descending and taxiing stages until the engine or the power is turned off. By using two cameras, stereo vision has a function similar to human eyes and can obtain 3D information on the targets. Stereo vision guidance system mainly consists of image capture, aircraft detection and tracking, and localization. As shown in Fig. 2, the detection algorithm extracts a pair of pixel points (*x*_*l*_, *y*_*l*_) and (*x*_*r*_, *y*_*r*_) from the captured sequential images, while the localization algorithm integrates the calibration data, a pair of detected pixel points and the feedback angles (*P*_*l*_, *T*_*l*_) and (*P*_*r*_, *T*_*r*_) of pan–tilt units into calculating the spatial coordinates at each time step. Mathematical models of the stereo localization were developed and illustrated in [9] at length.

## ROS-based detection algorithm

The ground stereo vision guidance system enables the UAV autonomy during takeoff-and-landing phases. As shown in Fig. 2, target detection is the first step and a kernel factor in the ground vision-based guidance. The detection algorithm aims at finding the flying vehicle’s coordinates from the captured sequential images. In the previous works [6–9], both corner-based and skeleton-based methods were employed into target detection for the ground stereo vision system. Typically, a skeleton-featured detection algorithm, namely Chan–Vese model, is considered and implemented in the ROS environment. Such an open-source implementation definitely draws attentions and technical supports from interested researchers. Advanced or newly developed detection algorithms are more smoothly fused into the ground stereo system.

### Skeleton-featured detection algorithm

The skeleton or edge is an important feature in images. The Chan–Vese model [19] is a geometry-driven active contour model that fuses both curve evolution and level set theories. To some extent, it can be expressed as zero level set of level set function indirectly.

Since the skeleton is a scale-, gray- and rotation-invariant feature, the Chan–Vese model-based detection possesses adaptability to object geometry or topology evolving. Therefore, the Chan–Vese detection is potentially suitable for all the ground vision-captured aircraft images, regardless of approaching, landing and taxiing on the runway.

The employed Chan–Vese approach is a kind of geometric active contour models. Although improper initial outline may lead to local minimum, the continuous movement of cooperative target can figure it out by estimating target’s position according to target movement characters. Combining target’s shape transformations with movement characters greatly improves the object detection accuracy. At the same time, the accuracy and efficiency of extraction will be improved with the development of image segmentation based on the theory of geometric active contour model.

Level set method increases the problem’s dimension to be higher. For example, a plane curve *C* is implicitly expressed as a same-value curve of three-dimensional continuous functional surface $\varphi (x,y,t)$, which is called level set function.

The Chan–Vese image segmentation is presented as follows. At first, a regular closed curve is given as the assumed original boundary. The closed curve iteratively evolves by numerically solving partial differential equations. Finally, it will converge to the target boundary.

The energy function *F*^*MS*^(*C*) of the Chan–Vese model is defined as:

1 $$\begin{aligned} F^{MS} (C) & = \mu L(C) + \nu area(insideC) \\ & \quad + \lambda_{1} \int_{insideC} {(u - c_{1} )^{2} } {\text{d}}x{\text{d}}y \\ & \quad + \lambda_{2} \int_{outsideC} {(u - c_{2} )^{2} } {\text{d}}x{\text{d}}y \\ \end{aligned}$$

where *C* is the ranging closed curve and *u* is matrix of the image. *μ* and *v* are coefficients. *c*_1_ and *c*_2_ are average pixel intensity values of inside and outside regions of the closed curve, respectively. Therefore, (*u* − *c*_1_)^2^ and (*u* − *c*_2_)^2^ can be treated as the pixel intensity values’ variance matrixes of inside and outside region. *L*(*C*) is the length of closed curve. A closed curve *C* needs to be found to minimize the energy function *F*^*MS*^(*C*) which is the final contour of segmentation.

Calculate a closed curve which minimizes the value of *F*^*MS*^(*C*) with the level set method. The level set function $\varphi (x,y)$ can be written as:

2 $$\varphi (x,y)\left\{ {\begin{array}{*{20}l} { = 0} \hfill & {(x,y) \in C} \hfill \\ { = d > 0} \hfill & {(x,y) \in insideC} \hfill \\ { = d < 0} \hfill & {(x,y) \in outsideC} \hfill \\ \end{array} } \right.$$

where *d* is the minimal distance between point (*x*_0_*, y*_0_) and the contour of closed curve *C*. The zero level set function which is the closed curve *C* can be written as:

3 $$\varphi (x,y) = 0$$

The Euler–Lagrange function of Chan–Vese model can be expressed as:

4 $$\left\{ \begin{array}{l} \frac{\partial \varphi }{\partial t} = \delta_{\varepsilon } (\varphi )\left[ {\mu \nabla \cdot \frac{\nabla \varphi }{|\nabla \varphi |} - v - \lambda_{1} (u - c_{1} )^{2} - \lambda_{2} (u - c_{2} )^{2} } \right] \hfill \\ \varphi (0,x,y) = \varphi_{0} (x,y) \hfill \\ \end{array} \right.$$

where $\varphi_0 (x,y)$ is the initial level set function which is usually set to be a circle in consideration of computation complexity of parameter *d*. $\varphi (x,y)$ evolves as time *t* pass. Iterations exit until $\partial\varphi/\partial t$ is closed to 0.

### ROS-based implementation

**Fig. 3:**
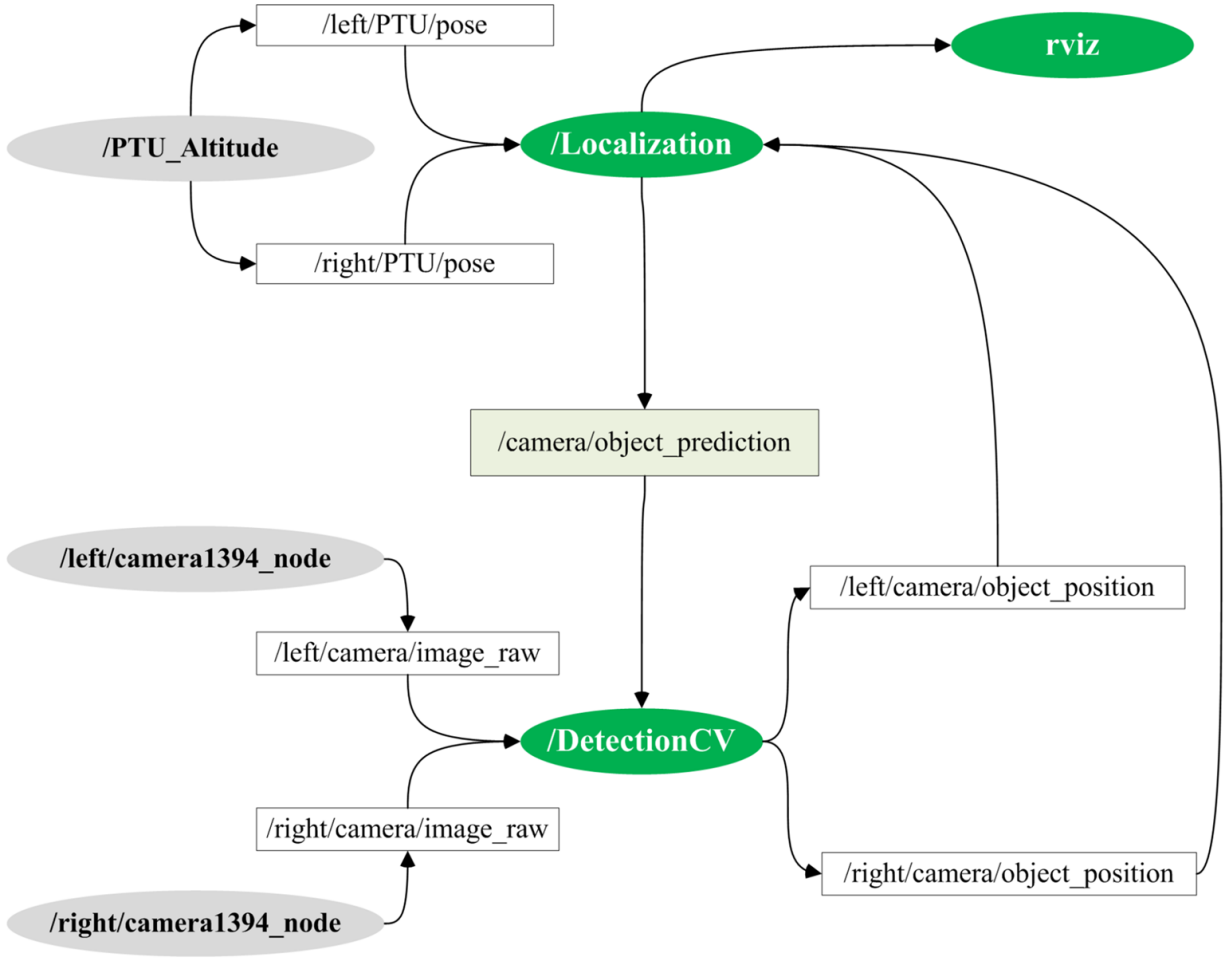
Logical and informational graph of the ROS-based detector. The *green* nodes represent the hardware PTUs and cameras in the ground system. The *green* nodes of  and  are kernel software modules. The input-and-output mapping of each node is given as well

The skeleton-featured Chan–Vese detection algorithm is implemented in the ROS *indigo* version and published as an open-source ROS package. Figure 3 presents the ROS node connection and topic communication topology when the ground stereo guidance system works.

As for the ROS-based implemented localization of flying aircrafts, the package is composed of two main software modules, namely  and  in Fig. 3. The ROS node  automatically detects the pixel coordinate of the flying aerial vehicle within the captured sequential images, while the other node  calculates the three-dimensional spatial coordinate by using the calibration data and the detected image coordinates. The  and  nodes provide the raw images captured by the left and right cameras. The  and  nodes provide the raw present states of the left and right PTU devices. The open-source detection package can be downloaded from the *github* Web site. Once appropriately configured in the ROS environments, the Chan–Vese detection package is run as the following steps.

- **Step 1:** Run the multiple cameras driver to publish the captured images, and find the ports of cameras (port1 and port2).
- **Step 2:** Run the PTU states publishing nodes.
- **Step 3:** Run the Chan–Vese detection node.
- **Step 4:** Run the stereo vision localization node.

## Experiments and discussion

The outdoor flight experiments are performed to collect the images, D-GPS data for the algorithm testing and parameter tuning. Simultaneously, the experiments demonstrate the usage and feasibility of the developed open-source ROS package.

### Algorithm demonstration

**Fig. 4:**
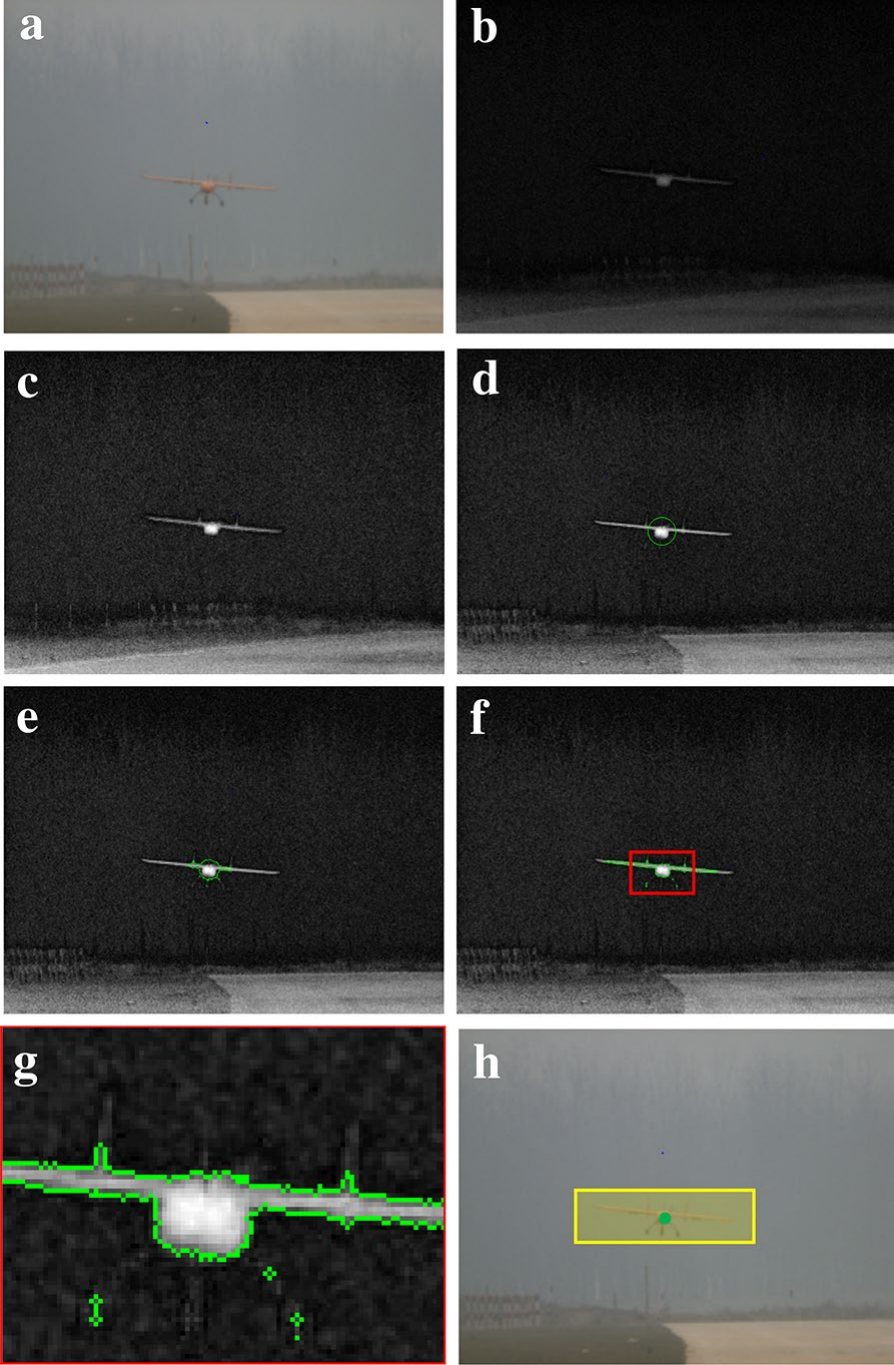
Object detection process of one selected image. **a** Original image, **b** S-channel component, **c** histogram equal image, **d** segmentation with the green contour as *φ*(*x*, *y*) = 0, **e** image with the first iteration, **f** image with the 20 iterations, **g** focused image within the *RED rectangle* and **h** detected pixel point shown at the original image

According to the skeleton-featured detection workflow, one frame of landing images is chosen to demonstrate how the processing runs. Segmentation procedures and results with the Chan–Vese algorithm are shown in Fig. 4. The *S*-channel component is extracted from the original image and equally histogram then. Segmentation is iteratively made on the transformed image. Images at typical iterations are given in the figure, e.g., the first and 20th iteration.

### Workflow of data-driven detection

In this study, a synthetic data-driven scheme is proposed to promote flying object detection algorithms. We concentrate on target checking and tracking on the UAV landing image sequences from the ground stereo vision guidance system. A manual interactive system is established to collect the aircraft coordinates in the sequential images, and moreover, datasets are constructed for training and evaluating various detection algorithms.

**Fig. 5:**
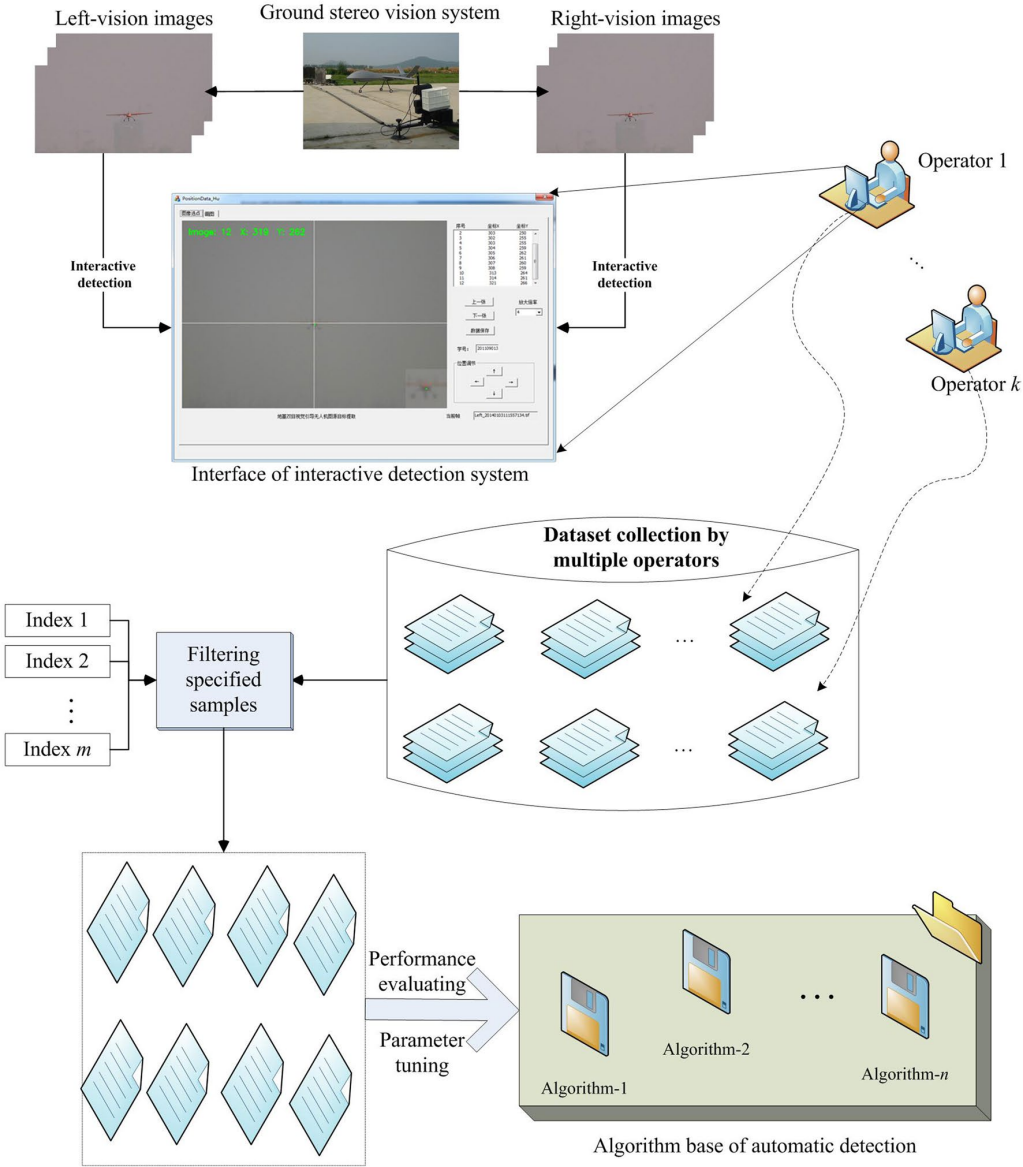
Workflow of data-driven detection on the landing sequential images. An interactive system enables manual collection of the aircraft coordinates in the image sequences. The constructed dataset is suitable for training and evaluating various automatic detection algorithms

**Fig. 6:**
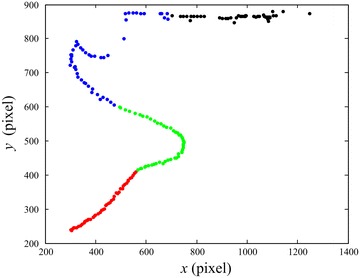
Sequential coordinates of manually detected UAV points. The manually detected pixel coordinates are exhibited within one specified UAV landing experiment

In the past works, a large number of sequential landing images have been captured in the runway-mode experimental flights [6, 7, 9]. The corresponding PTU, D-GPS and onboard sensor data are simultaneously recorded then. These data are usually obtained by human-in-loop or GPS-guided landing experiments, but they facilitate algorithm design and testing, performance evaluation, scheme comparison and so on. Figure 5 demonstrates the interactive system functions, such as image loading, zooming in and out, coordinates extracting, attribute registering, mistake correcting, dataset auto-saving and analyzing. Eventually, the evaluation dataset is constructed by multiple volunteers’ operations on the sequential images. Stochastic analysis methods are designed to generate the reference coordinates for evaluation on various automatic detection algorithms. Within the data-driven scheme, the manually detected pixel coordinates of sequential stereo images are recorded and classified in terms of landing experiments. As shown in Fig. 6, all the dotted points are transformed into the original image coordinate.

### Localization effectiveness

**Fig. 7:**
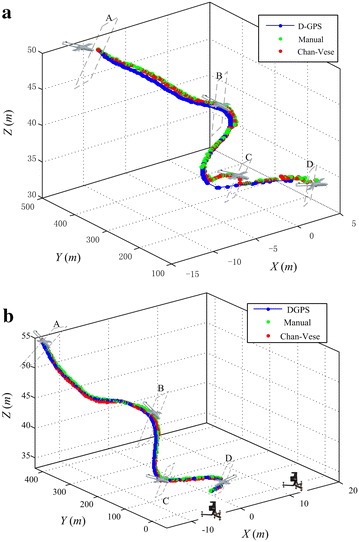
Localization accuracy comparison and analysis. The manual interactive and Chan–Vese automatic modes are simultaneously shown with the D-GPS data as the reference. **a** Landing trajectory of Case 1. **b** Landing trajectory of Case 2

Accuracy plays an important role in flying aircraft localization particularly within the autonomous landing stage. In the flight experiments, the D-GPS, manual detection and Chan-Vese automatic detection results are recorded, respectively. As shown in Fig. 7, the Chan–Vese detection algorithm possesses a mostly equivalent performance with the manual detection. Over 100 flights have been conducted in the Changsha Moon Island since the ground guidance prototype was implemented [9]. Three-mode localization trajectories are simultaneously exhibited in two specified representative cases. At the approaching stage starting from ‘A,’ localization accuracy is no obvious difference between all the three modes. Sequential images are captured with the blue or gray sky as the background, so the aircraft detection is much more accurate. When the aircraft approaches ‘B,’ the trees arise in the background and the detection is to some extent not as accurate as before. At the descending stage of ‘C,’ the maximum localization errors arise because the aircraft partially flies out of the camera view and some parts cannot be included in the captured images. At the taxiing stage of ‘D,’ higher accuracy is achieved again.

### Real-time feature analysis

The real-time property is another key factor in practical applications. In particular, it is much cared since the Chan–Vese segmentation is iteration-mode numerical solution on partial differential equations. As shown in Table 1, the control computer is with 2.80 GHz CPU and 6GB RAM, and the captured image size is 720 × 576. The time cost is listed in Table 1. The time-consuming of the Chan–Vese detection algorithm is 157 ± 10 ms. Such real-time feature is hardly acceptable for high-speed unmanned aircrafts, so other approaches are considered for detection speedup, such as CPU-GPU hybrid processing, predictive region of interest (ROI) and scale-space framework. These approaches shall be concentrated on for potential applications.

**Table 1:** Real-time feature of Chan–Vese detection algorithm

Parameter	Value	Unit
CPU	2.8G	Hz
RAM	6G	Byte
Image size	720 × 576	Pixel
Number of landing flights	79	–
Average frames per flight	132	Frame
Average time cost per frame	157 ± 10	ms
Minimum time cost for one frame	149	ms
Maximum time cost for one frame	175	ms

## Concluding remarks

Ground vision-aided guidance is demonstrated as an effective approach for runway-mode UAV autonomous landing. Compared with the onboard scheme, the developed ground vision system has the necessary processing power and greater computation capacity and furthermore rids the need for individual aircrafts to carry such equipment. Truth be told, the ground vision system has potential pitfalls as well. It has a limited distance and scope to make the first catch of flying aircrafts and is limited to weather conditions significantly. Furthermore, the instrument landing system (ILS) has already been around for decades of years and is deployed in almost every airport and manned airplane. That system with incredible accuracy is reported precise enough to allow landings in essentially zero visibility. Generally, its practical application is restricted to commercial passenger airports for the expensive consumption, inconvenient deployment and professional operations. The ground vision-based system can be modularly assembled and practically deployed for low-cost unmanned aircrafts. From the engineering point of view, the ground system can not only be developed as an effective supplement to the ILS in the high-level airports, but also make low-cost substitutes of ILS within specified scenarios.

In this article, open-source ROS implementation is employed into the ground stereo guidance system. This open scheme definitely enriches technical innovations from numerous interested researchers. Newly developed detection algorithms can be conveniently employed into the flying object detection. One representative of the Chan–Vese approach is considered and demonstrated in the ROS *indigo* version and published in the *github* Web site. The detection approach aims at locating the flying aircraft coordinates in the captured sequential images of the ground stereo vision guidance system. The running operators are given at length in the ROS-supported platform. Meanwhile, a data-driven interactive scheme is constructed since object detection in cluttered scenes requires large image collections with ground truth labels [20, 21]. Collection and use of annotated images play an important role in training and evaluation of detection approaches. Experimental comparisons are made by using the collected datasets, including the stereo sequential images, PTU angles, D-GPS positions and other flying states from onboard sensors. Results validate the effectiveness and generality of the published Chan–Vese detection ROS *indigo* package.

The open-source mode follows the present tendency in this field to draw more attentions, inspiration and contribution from online users. Furthermore, the annotated images and spatial extents should make positive effects on detection algorithm training and parameter optimization in the following researches.
